# Supplementary material for: Engineered symbiotic bacteria interfering Nosema redox system inhibit microsporidia parasitism in honeybees
Source: Nat Commun. 2023 May 20;14:2778. doi: 10.1038/s41467-023-38498-2 (PMC10199888; doi:10.1038/s41467-023-38498-2)
Supplement: Supplementary file 1 — Supplementary Information [file 41467_2023_38498_MOESM1_ESM.pdf]

# Supplementary Information

Engineered symbiotic bacteria interfering *Nosema* redox system inhibit microsporidia parasitism in honeybees

Lang H et al.

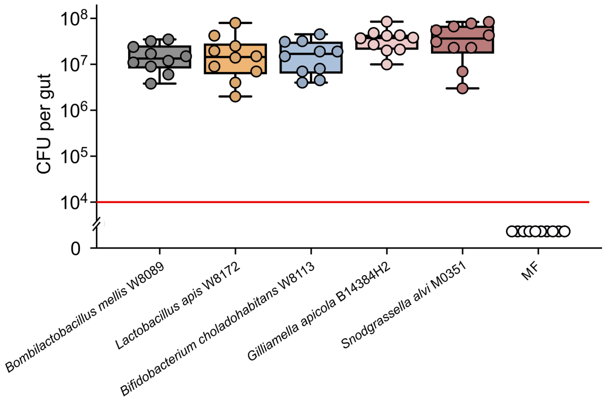


**Supplementary Fig. 1.** The graph showing the total CFU per gut estimated by bacteria culture for MF honeybees and honeybees mono-colonized with *B. choladohabitans* W8113, *B. mellis* W8089, *L. apis* W8172, and *G. apicola* B14384H2. Box plots indicate the median (middle line), 25th, 75th percentile (box), and 5th and 95th percentile (whiskers). n=10 biologically independent replicates.

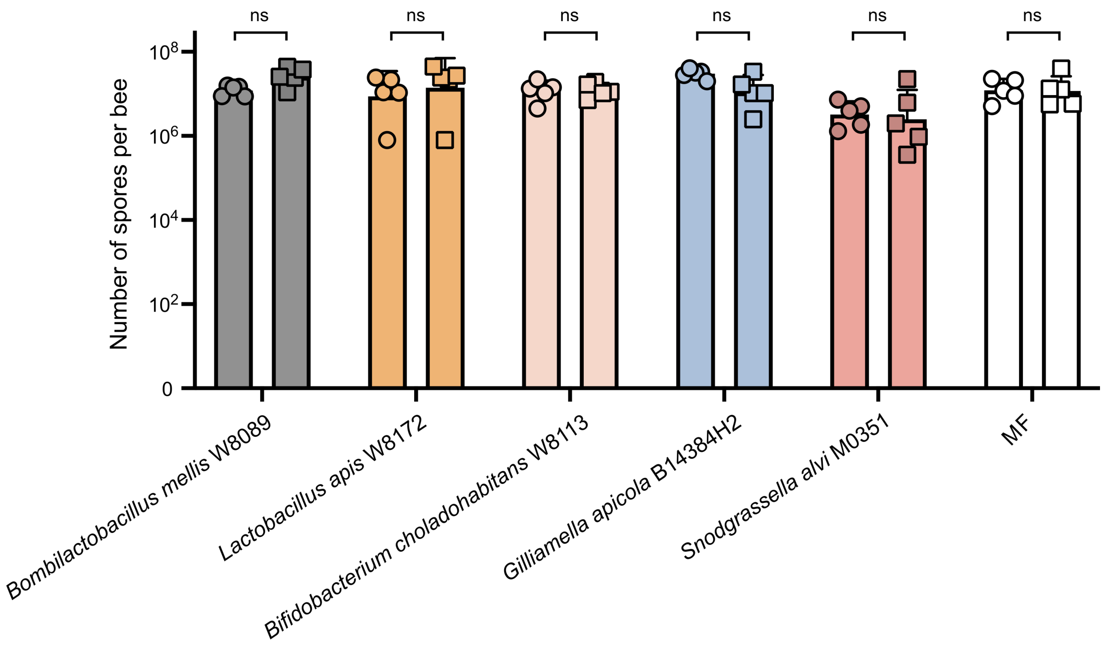


**Supplementary Fig. 2.** Absolute abundance of *N. ceranae* spores in the midgut 10 days post-infection for each colonization group. The shapes in the bar chart correspond to two independent source cup cages. Statistical analysis was performed by the two-sided Mann-Whitney U test, n = 5 bees for all groups. ns, not significant. Error bars represent mean SD.

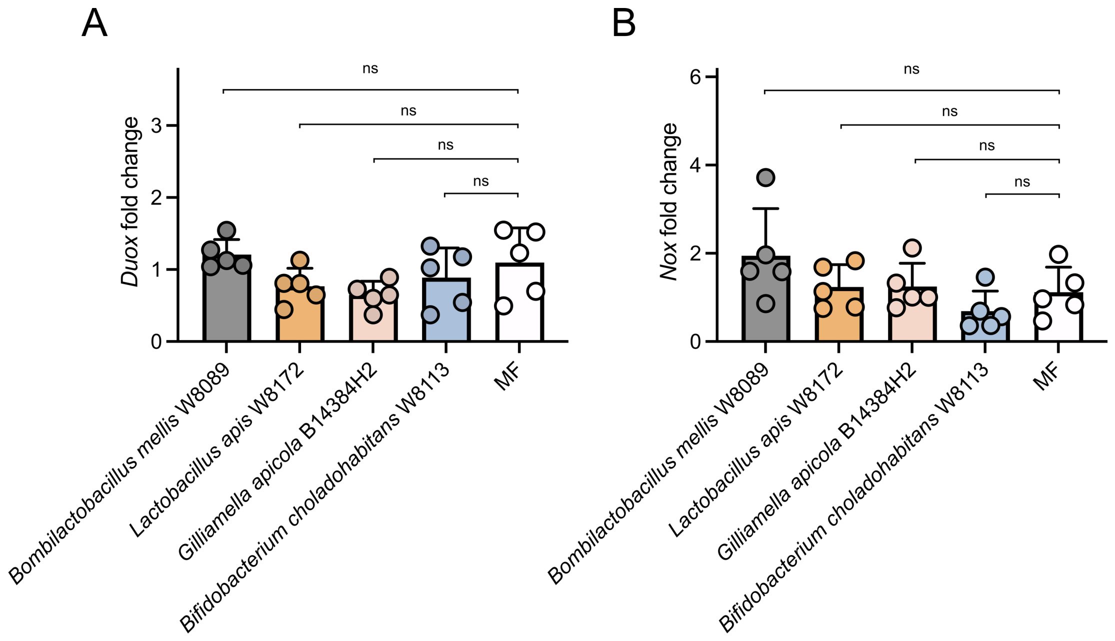


**Supplementary Fig. 3.** The expression level of the *Duox* (A) and *Nox* (B) genes in the midgut following *B. choladohabitans* W8113, *B. mellis* W8089, *L. apis* W8172 and *G. apicola* B14384H2 colonization. No statistical significance between each colonization group and the MF group tested by the two-sided Mann-Whitney U test (n = 5 bees for all groups). ns, not significant. Error bars represent mean SD.

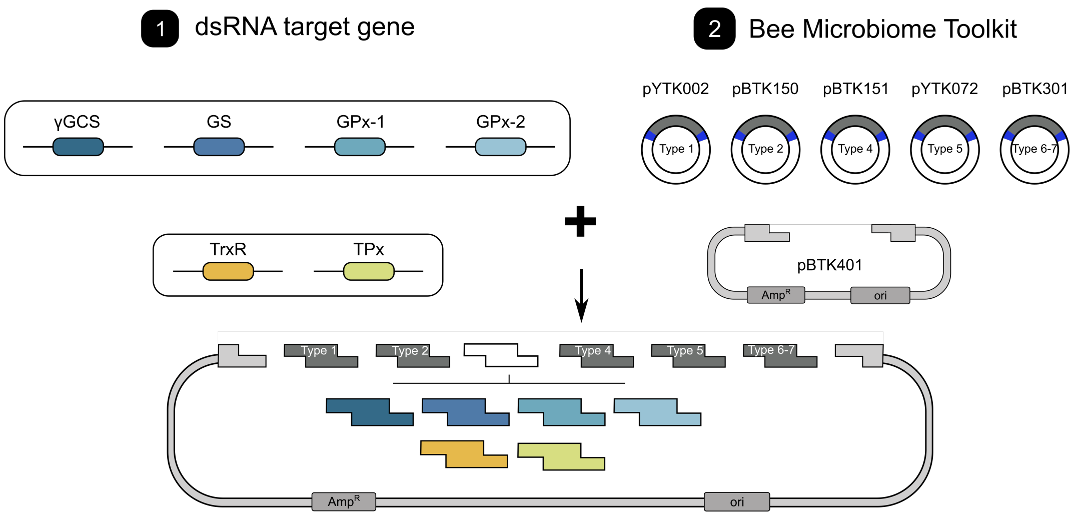


**Supplementary Fig. 4.** Schematic illustration of the Golden Gate assembly strategy for constructing plasmids expressing targeted dsRNA.

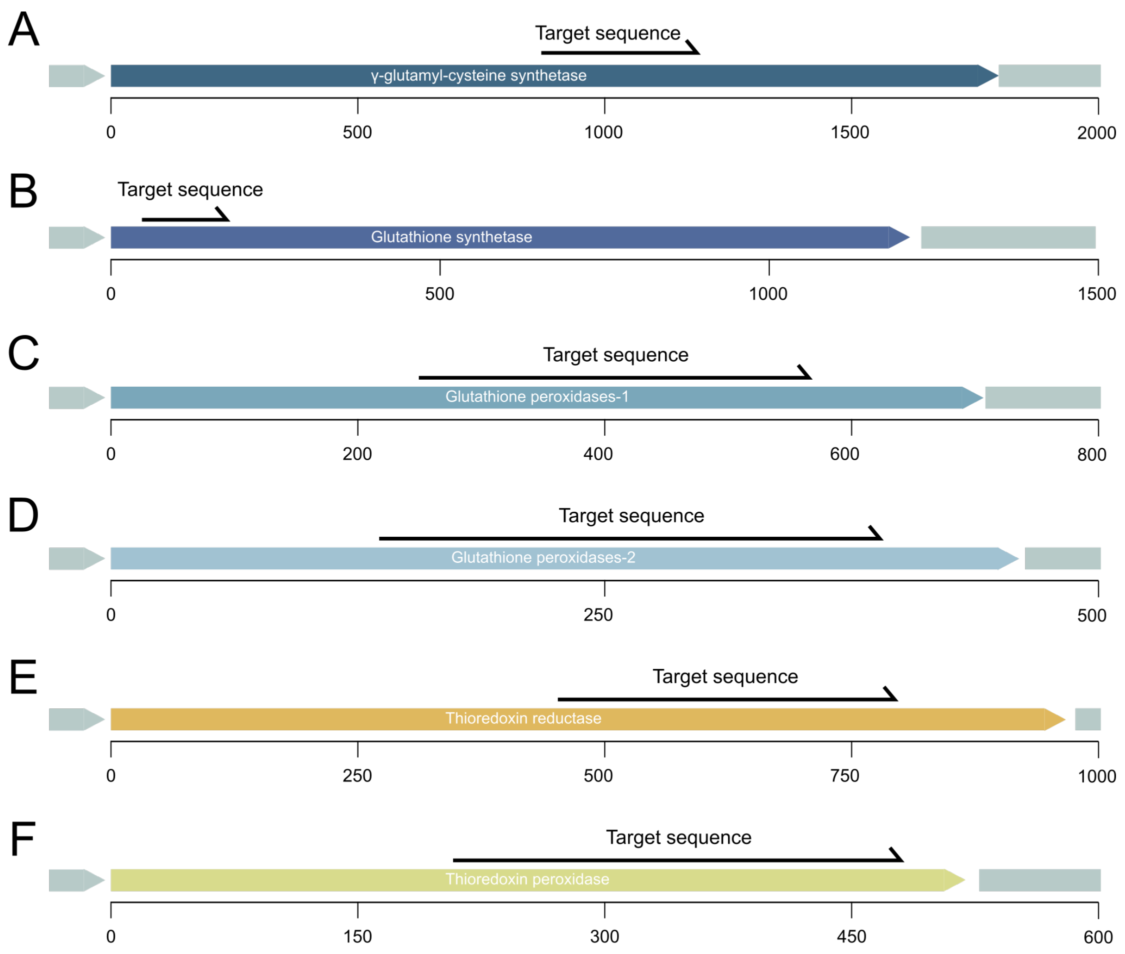


**Supplementary Fig. 5.** Design of *N. ceranae* targets. This diagram shows the overall gene organization for *N. ceranae* γ-glutamyl-cysteine synthetase (A), glutathione synthetase (B), Glutathione peroxidases-1 (C), Glutathione peroxidases-2 (D), thioredoxin reductase (E), and thioredoxin peroxidase (F). The targeted region in each gene is denoted, and the designed target sequences are listed in Supplementary Table 4.

**Supplementary Table 1**. List of bacterial strains.

| **Species and strain** | **Source** | **Accession No.** |
|---|---|---|
| *Escherichia coli* MFD*pir* | 1 | / |
| *Escherichia coli* DH5α | Vazyme | / |
| *Snodgrassella alvi* M0351 | 2 | JACFSG000000000 |
| *Bifidobacterium choladohabitans* W8113 | 2 | JACFQZ000000000 |
| *Bombilactobacillus mellis* W8089 | 2 | JACFRJ000000000 |
| *Lactobacillus apis* W8172 | 2 | JACFPG000000000 |
| *Gilliamella apicola* B14384H2 | 2 | JACFQC000000000 |

**Supplementary Table 2**. Plasmid list.

| Name | Use | Source |
|---|---|---|
| pBTK501 | Constitutive GFP | 1 |
| pYTK002 | Type 2 YTK/BTK connector sequence part plasmid | 1 |
| pYTK072 | Type 5 YTK/BTK connector sequence part plasmid | 1 |
| pBTK301 | Type 6-7 BTK bridge connector sequence part plasmid | 1 |
| pBTK401 | Type 8 origin of replication and origin of transfer plasmid | 1 |
| pBTK150 | Type 2 BTK part: terminator, CP25 promoter, no RBS | 3 |
| pBTK151 | Type 4 BTK part: reverse CP25 promoter, terminator, no RBS | 3 |
| pYTK001_T1T2 | Insulated part vector with flanking terminators | 3 |
| pDS-GFP | Control dsRNA GFP | 3 |
| pDS-γGCS | dsRNA target γGCS | This Study |
| pDS-GS | dsRNA target GS | This Study |
| pDS-GPx-1 | dsRNA target GPx-1 | This Study |
| pDS-GPx-2 | dsRNA target GPx-2 | This Study |
| pDS-TrxR | dsRNA target TrxR | This Study |
| pDS-TPx | dsRNA target TPx | This Study |

**Supplementary Table 3**. Primer list.

| Purpose | Gene | Gene Symbol in NCBI | Forward | Reverse | Source |
|---|---|---|---|---|---|
| qPCR | β-tubulin (*N. ceranae*) | AAJ76_300077627 | AGAACCAGGAACGATGGAGA | TCCTTGCAAACAATCTGCAC | 4 |
| qPCR | Duox (*A.mellifera*, Amel_HAv3.1) | LOC551970 | CCCTAATAGCCCTCGTGAAC | GAGCATTCTCATAACGTGTG | 5 |
| qPCR | Nox (*A.mellifera,* Amel_HAv3.1) | LOC408451 | ACCTGCTCTGTTTGGTCTCG | ACATCCACCACGTCGAACAA | This study |
| qPCR | γ-glutamyl-cysteine synthetase (*N. ceranae,* ASM18298v1) | AAJ76_1100057370 | AACAGGCGAGGGAAGAAACC | CAACCCATACCTTGGCCCAT | This study |
| qPCR | Glutathione synthetase (*N. ceranae*) | AAJ76_600034116 | TGGGAGTTTAATCGTTCTTGATGG | TCTAAACCGCCATTGCCAGA | This study |
| qPCR | Glutathione peroxidase (*N. ceranae*) | AAJ76_3500027152 | GGCAAATCCACATGTAGAAGC | TCTGCTATAGACTACAAAGACAAGA | This study |
| qPCR | Glutathione peroxidase (*N. ceranae*) | AAJ76_3500027978 | TGCAGTTGATTCAAAACAGTCAC | AGGCCTTTGTCATAAAATTCGTCT | This study |
| qPCR | Thioredoxin reductase (*N. ceranae*) | AAJ76_5800012528 | GTGGTCCTGCAGCCTATTCT | TACTTCCTACCATCCCGCCT | This study |
| qPCR | Thioredoxin peroxidase (*N. ceranae*) | AAJ76_280004776 | TGATTTTACTTTTGTGTGTCCAACT | CACCGTCCTGTCTTGGGAG | This study |
| qPCR | SSU rRNA (*N. ceranae*) | AAJ76_1700061205 | GTCGCTATGATCGCTTGCC | TATTGTAGAGAGGTGGGAGATT | 6 |
| RNAI_Type3 | γ-glutamyl-cysteine synthetase (*N. ceranae*) | AAJ76_1100057370 | CGGTCTCATATGCCAAAAAGCAGGTGTTCTCCA | TGGTCTCAGGATGGGAGTTGCTTGCATTTCCAT | This study |
| RNAI_Type3 | Glutathione synthetase (*N. ceranae*) | AAJ76_600034116 | CGGTCTCATATGGCTAGAGCGGTGGGTTTAGT | TGGTCTCAGGATACCAAATGATACAAAACACTGTGAA | This study |
| RNAI_Type3 | Glutathione peroxidase (*N. ceranae*) | AAJ76_3500027152 | CGGTCTCATATGGCGCACTTCTAGACGATCTCC | TGGTCTCAGGATACATGTGGATTTGCCAAGAGT | This study |
| RNAI_Type3 | Glutathione peroxidase (*N. ceranae*) | AAJ76_3500027978 | CGGTCTCATATGTTCCTTGTTCGCAATTTTTAAACCA | TGGTCTCAGGATATAACCTCTGTTGGTCCATATCGC | This study |
| RNAI_Type3 | Thioredoxin reductase (*N. ceranae*) | AAJ76_5800012528 | CGGTCTCATATGAGGAGGAGACTCTGCTATGGA | TGGTCTCAGGATCGCAGTCGTACAATCCTCGT | This study |
| RNAI_Type3 | Thioredoxin peroxidase (*N. ceranae*) | AAJ76_280004776 | CGGTCTCATATGACGTTGTCAGTGATAGGAAGGA | TGGTCTCAGGATACACATGATCCATTGCGTCCA | This study |
| ds_RNAi | γ-glutamyl-cysteine synthetase (*N. ceranae*) | AAJ76_1100057370 | TAATACGACTCACTATAGGGTTGGATTTCTGGGCAACGGT | TAATACGACTCACTATAGGGCGAACCTCGTCAGCAAAAGG | This study |
| ds_RNAi | Thioredoxin reductase (*N. ceranae*) | AAJ76_5800012528 | TAATACGACTCACTATAGGGAGGAGGAGACTCTGCTATGGA | TAATACGACTCACTATAGGGCGCAGTCGTACAATCCTCGT | This study |
| *Nosema apis* | SSU rRNA (*N. apis*) | U97150 | CCATTGCCGGATAAGAGAGT | TATATTTATTGTATTGCGCGTGCT | 7 |
| *Nosema ceranae* | SSU rRNA (*N. ceranae*) | DQ486027 | AGAAACTACAACAGCATCACTGGGA | AGTGAATATTCCAATTCCCAACGACTT | 7 |

**Supplementary Table 4**. Target sequences of dsRNA.

| ID | Use | Length (bp) | GC % | Sequence Source |
|---|---|---|---|---|
| **pDS-γGCS** | γGCS knockdown | 339 | 35 | This Study |
| CCAAAAAGCAGGTGTTCTCCATTTGATTTATTTATTTCTAATGACGCTAGAAATTTAGACAAGTACAACGATACAAATCCACCG<br>ATCCACGTGCCGATTTTTAATAAATTAATAAAGAAAGGTGTTGATGAAAAATTGTCGCGACATGTAGCATCTTTATTTATTCGC<br>GATCCTATAGTCTCATATGACGAAACAGATGAAAGCACATTTGACGATTTTGAAAACATACAAAGTTCAAATTGGAGATCTGTA<br>AGGTTTAAAGTACCAACAGAAAGTTCTGATAAAGATTTAAGAGGATGGAAAGTTGAAGTTCGTCCTATGGAAATGCAAGCAAC<br>TCCC | | | | |
| **pDS-GS** | GS knockdown | 330 | 27 | This Study |
| GCTAGAGCGGTGGGTTTAGTAAATCCATCTACAGGAAACATACTAAATATAACTATAAGACCCTCTAAAATATCAAGATTACAT<br>TTTTTATCGATAAAAAATTTACAATTAAAACTTAATTTACTGTACTACAATCTAAGCAAAGACTTAGATTCATACACTTTTGATGA<br>ACCAATGTACAAATTTTTGCACTCGATTTACACTGAAAAAAAAATGGTAAAAAAAGACAATATAGTAGCACTTTATATTAGATCT<br>GATTATTTATTAGACGGTGACCAATATAAACAAGTAGAAATTAATACTATTTCACAGTGTTTTGTATCATTTGGT | | | | |
| **pDS-GPx-1** | GPx-1 knockdown | 348 | 28 | This Study |
| ACATGTGGATTTGCCAAGAGTAATCTTTCTATACTAGCTGATATTATGACACTGTATAAAGAATATGGCCTTAAAATATTGATC<br>TTTCCTTGTCTGCAATATTCAAAAGATGATACTGATTTACTTAAAAAGATGTATAACTTAATAACAGAATATTCTGATGATTTTA<br>CAGTATTTAGTGATATTAATTTGGTCGGTAAAAATATACATCCTGTTTATAAGCATATTGTAAGGTATAGAAAAGAGTTTGTAG<br>GAGATTTTATAAAATGGAATTTCGCAAAATTTATTGTTAATGAATACGGTGAAATAGTAAAAATTTTTGAACCAGGAGATCGTC<br>TAGAAGTGCGC | | | | |
| **pDS-GPx-2** | GPx-2 knockdown | 250 | 30 | This Study |
| TTCCTTGTTCGCAATTTTTAAACCAGGAATCTGGTGATATTGAAACTATTCGCAATTATGCGCATTCTATTTCTGATAAATTTAT<br>CGTATTTGACAAAGTTAATGTATTTGGATCTCAAAAAGATCCTGTTTTTAAGTATTTAACAGACAACAGTTCAGAAAGTTTTTTT<br>AAGTTTGTAAAATGGAACTTTACAAAATGGGTTGTTAAAGATGGAAAGATTTTAAAGCGATATGGACCAACAGAGGTTAT | | | | |
| **pDS-TrxR** | TrxR knockdown | 349 | 28 | This Study |
| AGGAGGAGACTCTGCTATGGAAGAAGTCTTATTTTTATCTAAAATATGTTCTAAAGTTTATTTAATTCATCGAAGAAATGAATTT<br>AGAAGCAGGAAGGATAAATTAGAAGAAGTAAAGAAGACAAAAAATATAGTTATAGTTACACCATATAATCTAAAATCTGCCCAT<br>GGAATTGATAAATTAGAATATATTATCCTTGAAAATGGGGCAGAAACTAAAAAATTAGAAGTAGATGGATTGTTCTTTGGTATC<br>GGTCATACACCTAATACTCAATTTTTAGAAGAAAATTTTTTACATGTTTTAGACAAAGATAAGTTTATTAAAGTAAACGAGGATT<br>GTACGACTGCG | | | | |
| **pDS-TPx** | TPx knockdown | 330 | 37 | This Study |
| ACGTTGTCAGTGATAGGAAGGAAGAGTTTATTAAACGCAATGTTGTTGTGTTTACAATTTCTAAAGATTCCGCATACAATCATC<br>AAGCATGGGCTAAACTCCCAAGACAGGACGGTGGCGTAGAAGGAATACAATGGCCAATGTTGGCAGATAAGAATGCTCGTT<br>TAAGTCGACAGTTTGGTTTGTATGATGATGAGGAAGATATCACCAAAAGGGCTACTGTAATGATTGATGTTTCTGGAAATGTG<br>TTTAATATTTCTATCTATCATGAAAAAATAGGAAGAAATGTTGATGAAATTTTGAGGTTATTGGACGCAATGGATCATGTGT | | | | |

## References


1. Leonard SP*, et al.* Genetic Engineering of Bee Gut Microbiome Bacteria with a Toolkit for Modular Assembly of Broad-Host-Range Plasmids. *ACS Synth Biol* **7**, 1279-1290 (2018).

2. Wu J*, et al.* Honey bee genetics shape the strain-level structure of gut microbiota in social transmission. *Microbiome* **9**, 225 (2021).

3. Leonard SP*, et al.* Engineered symbionts activate honey bee immunity and limit pathogens. *Science* **367**, 573-576 (2020).

4. He N*, et al.* RNA Interference-Mediated Knockdown of Genes Encoding Spore Wall Proteins Confers Protection against Nosema ceranae Infection in the European Honey Bee, Apis mellifera. *Microorganisms* **9**, (2021).

5. Azzouz-Olden F, Hunt A, DeGrandi-Hoffman G. Transcriptional response of honey bee (Apis mellifera) to differential nutritional status and Nosema infection. *BMC Genomics* **19**, 628 (2018).

6. Forsgren E, Fries I. Comparative virulence of Nosema ceranae and Nosema apis in individual European honey bees. *Vet Parasitol* **170**, 212-217 (2010).

7. Schwarz RS, Evans JD. Single and mixed-species trypanosome and microsporidia infections elicit distinct, ephemeral cellular and humoral immune responses in honey bees. *Dev Comp Immunol* **40**, 300-310 (2013).
